# The Dual Role of Natural Peptides in Cancer Therapy: Anticancer and Immunomodulatory Perspectives

**DOI:** 10.32604/or.2026.078405

**Published:** 2026-06-16

**Authors:** Avetis Tsaturyan, Heghine Hakobyan, Kristina Hovsepyan, Anna Mkrtchyan, Tatevik Sargsyan, Raffaele Pastore, Germano Guerra, Giovanni N. Roviello

**Affiliations:** 1 Scientific and Production Center ‘Armbiotechnology’ NAS RA, 14 Gyurjyan Str., Yerevan, Armenia; 2 Institute of Pharmacy, Yerevan State University, 1 Alex Manoogian Str., Yerevan, Armenia; 3 Department of Medicine and Health Sciences ‘Vincenzo Tiberio’, University of Molise, Via F. De Santis, Campobasso, Italy; 4 Institute of Biostructures and Bioimaging (IBB) of the Italian National Council for Research (CNR), Area di Ricerca site and Headquarters, Via Pietro Castellino 111, Naples, Italy

**Keywords:** Natural peptides, anticancer therapy, immunomodulation, tumor microenvironment, angiogenesis inhibition, drug resistance, nanotechnology, personalized medicine

## Abstract

Cancer is regarded as one of the leading causes of death worldwide, despite the progress of traditional therapies. Chemotherapy, radiotherapy, and surgery are often accompanied by significant side effects and the development of drug resistance contributes to making the fight against cancer even more challenging, which clearly highlights the urgent need to develop new therapeutic molecular approaches. In this context, natural peptides were introduced into the pharmaceutical market in the last decade and have replenished the ranks of effective anticancer agents due to their structural diversity, biocompatibility, and ability to selectively target tumor cells. Natural peptides play a dual role, directly inhibiting tumor growth and proliferation by inhibiting angiogenesis and, conversely, exhibiting an immunomodulatory effect by enhancing the activation of T lymphocytes and natural killer cells and positively altering the tumor microenvironment (TME). The aim of this work is to critically evaluate the available literature on the anticancer and immunomodulatory activities of natural peptides and their potential use, both as monotherapy and in combination therapy, with particular attention given to issues of stability, bioavailability, and scalability of production. As highlighted throughout this review, a promising area is the integration of natural peptides with their synthetic derivatives and combining them with modern approaches such as nanotechnology and personalized medicine, which opens new avenues in cancer treatment.

## 1 Introduction

Despite significant advances in cancer diagnosis and treatment over recent decades, the disease remains one of the leading causes of mortality worldwide. International epidemiological data indicate that approximately 19–20 million new cancer cases are diagnosed each year globally, and more than 10 million deaths are attributed to cancer annually [1,2,3]. The most widely used approaches for cancer treatment include surgery, radiation therapy, and chemotherapy; however, each is associated with significant limitations, such as systemic toxicity, damage to healthy tissues, the development of drug resistance, and insufficient selectivity toward malignant cells [4,5,6]. Consequently, there is an ongoing search for novel therapeutic strategies that offer greater target specificity and reduced adverse effects. In this context, natural peptides have gained increasing attention in recent years as promising experimental anticancer agents [7,8,9].

Peptides are increasingly recognized as a promising class of anticancer agents owing to their structural diversity, inherent biocompatibility, and ability to selectively interact with tumor cells that are altered or under stress. Moreover, they are generally less prone to inducing multidrug resistance compared with conventional chemotherapeutic agents [9,10,11,12]. However, it is important to consider that cancer cells can still develop resistance through non-classical adaptive mechanisms. Several natural peptides and their derivatives have already been used in clinical practice or are currently being tested in different stages of clinical evaluation. These findings indicate that natural peptides may have substantial therapeutic potential in cancer treatment, appearing promising both as standalone agents and in combination with existing therapies.

A distinguishing feature of natural peptides is their dual mechanism of action. First, many peptides exert direct cytotoxic effects on tumor cells by disrupting cancer cell membranes, inducing mitochondrial dysfunction, inhibiting angiogenesis, and interfering with proliferative signaling pathways. Second, numerous peptides exhibit potent immunomodulatory properties. They enhance the activity of T lymphocytes and natural killer (NK) cells, stimulate cytokine production, and modulate the tumor microenvironment in ways that support antitumor immune responses [8,9,12,13]. In other words, natural peptides are bifunctional molecules capable of simultaneously inhibiting tumor growth and activating immune responses to help control neoplastic progression. 

Nonetheless, their practical application is limited by several challenges, including their low stability in biological environments, suboptimal bioavailability, potential cytotoxicity toward normal cells, as well as difficulties in large-scale production and high synthesis costs [14,15]. Recently, considerable attention has also been paid to the chemical modification of peptides and the creation of stable analogs, as well as the optimization of amino acid sequences and the improvement of their delivery systems, including those using nanotechnology. These strategies broaden the therapeutic applicability of peptides and enhance their clinical relevance. Today, the concept of personalized peptide therapy, based on individual tumor characteristics and the patient’;s immune status, is currently gaining increasing attention. Combined with nanomedicine, molecular design, and modern bioengineering technologies, peptides open new opportunities for the creation of highly selective and minimally invasive anticancer agents [16,17]. 

However, most published reviews of natural anticancer peptides focus primarily on their cytotoxic effects or specific aspects of peptide immunotherapy and vaccination. The dual anticancer and immunomodulatory activity of natural peptides, as well as the structural and functional characteristics of peptides facilitate the integration of their cytotoxic activity with the stimulation of innate and adaptive immune responses to remodel the tumor microenvironment. Even though these interrelated mechanisms can be exploited in the context of nanomedicine and personalized cancer therapy, several factors presently constrain peptide clinical application, including their stability, bioavailability, vulnerability to proteolytic degradation, issues that can be overcome by chemical modification of natural peptides and the use of nanotechnological delivery systems.

This work aims to provide a critical assessment of the existing literature on the anticancer and immunomodulatory properties of natural peptides, examining their potential application as single-agent treatments or in combination therapies, with particular focus on challenges related to stability, bioavailability, and scalability of production. 

### 1.1 Integrating Natural Peptides with Emerging Therapeutic Technologies

Importantly, while this review primarily concentrates on natural peptides, we also incorporated some information from the relevant research on synthetic peptide constructs. In fact, rational peptide design strategies [18,19,20,21], including amino acid substitutions, cyclization, lipidation, PEGylation, and the incorporation of non-natural amino acids, all can markedly enhance the pharmacological properties of the resulting peptide molecules [22,23,24]. At the same time, advances in computational design, directed evolution, and bioinformatics-based modeling have enabled the development of peptides with enhanced target selectivity, improved resistance to proteolytic degradation, and increased physiological availability. As a result, synthetic and chemically modified peptides are increasingly recognized as valuable complements to natural anticancer peptides, thereby broadening the spectrum of available therapeutic strategies. 

In this context, the rapid advancement in automated peptide synthesis [25,26,27], has significantly accelerated the development of peptide-based therapeutics. Concurrently, nanocarrier technologies—including liposomal and lipid-based systems [28,29,30,31], advanced nanostructured and encapsulation platforms [32,33,34,35], and smart polymer-based and sustained-release delivery systems for peptides and proteins [36,37,38] have markedly improved peptide stability, bioavailability, and controlled release, thereby facilitating the development of next-generation peptide-based anticancer agents [39,40]. In this context, the exploitation of both natural and synthetic peptide-based approaches signifies one of the most auspicious pathways for the progression of peptide oncotherapy in the age of personalized medicine. 

Beyond the rapidly expanding field of peptide-based anticancer strategies, contemporary biomedicine is simultaneously advancing across multiple complementary research directions. These include the growing interest in natural products as modulators of neurodegenerative pathways [41]. Significant advances are also being made in elucidating the mechanisms and global impact of intracellular parasitic infections [42], together with the refinement of precision-medicine approaches that integrate bioactive molecules, multi-omics datasets, and drug-repurposing strategies [43]. In parallel, researchers are developing peptides as new antimicrobial drugs [44] and investigating biomacromolecular interactions and antioxidant properties of newly engineered amino acid-based molecules. In particular, recent studies have focused on the development of advanced synthetic strategies for non-proteinogenic α-amino acids and their derivatives [45,46,47,48], while complementary research has explored their biological activity, molecular docking profiles, and interactions with target biomacromolecules [49,50,51]. Additional progress includes the design of nature-inspired covalent inhibitors targeting pathogenic and cancer-related proteins [52]. Further innovation is reflected in the valorization of dairy and fermentation side streams into functional food ingredients [53,54,55,56], as well as in the recovery and functional application of bioactive compounds from grape pomace [57,58] and other plant-derived residues [59,60,61,62,63]. In parallel, advances in analytical chemistry methodologies continue to support these developments [64]. Overall, these developments reflect the dynamic landscape of modern biomedicine and highlight how peptide-based anticancer and immunomodulatory strategies align with broader technological and therapeutic innovation. 

In this context, this review proposes that natural peptides represent the biological foundation for anticancer peptide development, whereas synthetic and chemically modified analogs serve as a rational extension designed to enhance their properties and facilitate clinical translation, as discussed in the sections below. While previous reviews have often treated anticancer activity and peptide immunotherapy as separate domains, this work uniquely integrates these functions under a single mechanistic framework. We define ‘natural peptides’ as native sequences derived from biological sources, which serve as foundational templates. Synthetic derivatives and nanotechnological approaches are discussed herein not as separate entities, but as essential rational extensions required to overcome the inherent pharmacological limitations of natural leads.

### 1.2 Methodology

The literature examined in this review was sourced via a focused search of Google Scholar, PubMed, Scopus, and Web of Science databases. The search strategy used combinations of keywords like “anticancer peptides”, “immunomodulatory peptides”, “tumor microenvironment”, “innate immunity”, “peptide nanocarriers”, “peptide vaccines”, and “personalized oncology” [7,8,9,15,16]. Research published from 1991 to 2025 was examined, focusing specifically on recent studies from 2018 to 2025 that illustrate the contemporary understanding of the dual anticancer and immunomodulatory properties of both natural and modified peptides [4,5,13,14,15]. 

The selection criteria for literature inclusion were: (1) original research and review articles published in English, (2) studies specifically focusing on peptides of natural origin or their direct synthetic derivatives, and (3) research highlighting dual anticancer and immunomodulatory activities. Articles focusing solely on non-peptide small molecules or those published in languages other than English were excluded to ensure the quality and thematic focus of this review.

## 2 Molecular and Structural Basis for the Anticancer and Immunomodulatory Action of Natural Peptides

As anticipated, natural peptides form a large and structurally diverse group of amino acid sequences that possess the unique ability to simultaneously suppress tumor cell viability and activate the anticancer immune response. This dual effect is explained by a few fundamental physicochemical and biological properties that distinguish peptides from traditional small-molecule chemotherapeutic agents. Thus, understanding structure-function relationships is key to developing new therapeutic strategies based on natural and synthetic peptides [65,66,67].

### 2.1 Physicochemical Properties Determining the Selectivity of Natural Peptides on Tumor Cells

Unlike conventional chemotherapy, which primarily targets rapidly dividing cells by inducing systemic DNA damage, often leading to severe side effects such as myelosuppression and hair loss, natural peptides exhibit a more selective mechanism of action. By targeting the unique biophysical properties of tumor cell membranes (e.g., negative charge and increased fluidity), peptides achieve a higher therapeutic index. This selectivity not only reduces off-target toxicity toward healthy tissues but also minimizes the risk of secondary malignancies often associated with traditional radiotherapy and alkylating agents [4,5,12,13,15]. Current data indicate that charge asymmetry, abnormal lipid organization, increased membrane fluidity, structural defects in repair systems, and tumor metabolism features, reflected in increased sensitivity to oxidative stress, play a key role. The following sections summarize the main contemporary models describing anticancer peptide (ACP) molecular selectivity [68,69].

#### 2.1.1 Increased Negative Charge of the Tumor Cell Membrane Is a Key Element of Primary Recognition

One of the most studied factors is the exposure of phosphatidylserine (PS) on the outer leaflet of the plasma membrane. In normal cells, PS is localized in the inner monolayer, and its external release is observed only during apoptosis. In tumor cells, the concentration of PS on the outer surface increases 3–7-fold [70]. This is accompanied by impaired expression of annexin V and dysregulation of flip-flop transport mechanisms, resulting in the formation of a large negatively charged surface region that is highly conducive to the binding of cationic anticancer peptides [7,71,72]. 

In addition to phosphatidylserine, heparan sulfates, chondroitin sulfates, highly sulfated mucins (MUC1 and MUC16), and sialylated glycolipids (GM3, GD2, and GD3) also contribute to the increased negative charge of tumor cell membranes. For example, GD2 and GD3 are frequently overexpressed in neuroblastoma, melanoma, and breast cancer, thereby enhancing electrostatic interactions with cationic and amphiphilic ACPs and contributing to tumor selectivity for certain membrane-active peptides. 

Current models describe ACP-membrane interactions as an initial electrostatic attraction followed by partial depolarization, lipid bilayer insertion, and pore formation, representing a critical early step in ACP-mediated cytotoxicity [73,74,75].

#### 2.1.2 Altered Membrane Lipid Composition and Increased Membrane Fluidity in Tumor Cells

Recent studies demonstrate that transformed cells undergo substantial alterations in the lipid composition of their plasma membranes, which significantly influence the biophysical properties of the membrane [76,77,78,79,80]. Cholesterol is a key structural component of the plasma membrane, contributing to membrane stability by promoting tight lipid packing and reducing fluidity and permeability. In various tumor types, including certain leukemias as well as lung and breast cancers, both the overall abundance and lateral distribution of membrane cholesterol are altered. 

In many highly motile or metastatic cancer cells, reduced surface cholesterol correlates with increased membrane fluidity, deformability, and structural disorder. This more loosely packed membrane environment facilitates the insertion of amphiphilic cationic peptides and promotes pore formation, consistent with mechanisms proposed in the carpet-like and toroidal pore mode [81,82,83]. In contrast, some tumors, such as prostate cancer, are characterized by an enrichment of cholesterol within lipid raft microdomains. Despite this local cholesterol abundance, experimental disruption or fragmentation of these rafts through cholesterol-modulating agents sensitize cancer cells to apoptosis and membrane-active compounds. Thus, both cholesterol depletion and destabilization of cholesterol-rich microdomains can enhance membrane susceptibility to ACP-mediated damage, albeit via distinct mechanisms [84,85,86]. 

Increased membrane fluidity and lipid disorder are further reinforced by structural and compositional changes characteristic of tumor cells. Cancer cell membranes often display an expanded surface area with numerous microvilli and a shift in phospholipid composition toward a lower proportion of saturated fatty acid chains. This reduction in lipid saturation decreases membrane order and stiffness, promoting dynamic and disorganized lipid packing. Computational and experimental modeling studies indicate that amphiphilic ACPs insert more readily into such fluid and disordered membranes, thereby facilitating peptide accumulation, membrane thinning, and pore formation [73,87,88]. 

As mentioned before, lipid rafts might also be important targets for some amphiphilic cationic peptides. In fact, certain peptides preferentially interact with raft-like membrane domains, depending on the membrane’s lipid composition and structural organization. Melittin, for example, has been shown to disrupt glycosphingolipid-rich microdomains containing GM1 and GM3. In contrast, dolastatin-derived peptides primarily alter cytoskeletal and microtubule dynamics, indirectly influencing raft-associated signaling pathways and membrane organization. 

Alterations in cholesterol content, lipid saturation, membrane architecture, and lipid-raft integrity can act synergistically to create a membrane ‘window of vulnerability’, thereby increasing the susceptibility of tumor cells to damage by ACPs [89,90,91]. These membrane alterations not only facilitate the initial interaction with anticancer peptides but also amplify the metabolic vulnerability of tumor cells, creating a context in which additional mechanisms, such as oxidative stress, become particularly effective.

#### 2.1.3 Increased Susceptibility of Tumor Cells to Oxidative Stress

Several natural anticancer agents, such as the amphipathic peptide melittin, lactoferricin-derived fragments like LfcinB25, and small molecules like perillyl alcohol (PA and second-generation PA-II derivatives), have been shown to raise the levels of reactive oxygen species (ROS) in tumor cells. In different models, these substances can cause mitochondrial dysfunction, lipid peroxidation, and the activation of the intrinsic caspase cascade, which eventually leads to apoptosis. 

Redox homeostasis is consistently disrupted in cancer. Tumor cells experience hypoxia, metabolic reprogramming toward aerobic glycolysis (the Warburg effect), and alterations in tumor-suppressor pathways such as those governed by p53. These factors collectively modify both the generation of ROS and the cellular antioxidant defenses. Many cancer cells operate near the threshold of their antioxidant capacity because key enzymatic systems, including superoxide dismutases (SODs), glutathione peroxidases (GPx), and catalase, are frequently dysregulated. In some tumors, these enzymes are upregulated to counteract elevated ROS, whereas in others they are downregulated. 

Under such conditions, even a modest additional increase in ROS induced by peptides or related agents can destabilize the redox balance, resulting in rapid oxidative damage to membrane lipids, mitochondrial dysfunction, and caspase-dependent cell death [92,93,94].

### 2.2 Key Structural Motifs Determining Biological Activity

**Figure 1:**
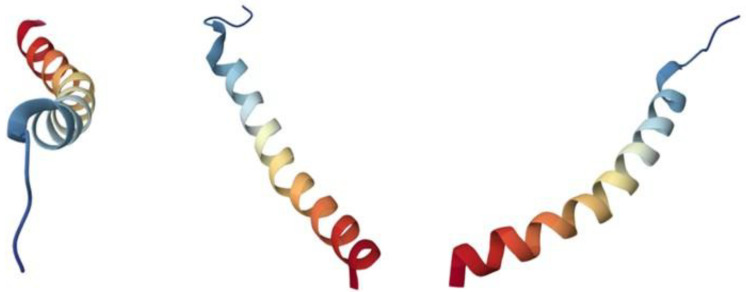
Three-dimensional structural views of the human antimicrobial peptide LL-37. The model shown here is derived from the publicly available structure deposited in the RCSB Protein Data Bank (PDB ID: 2K6O https://www.rcsb.org/3d-view/2K6O/0 [95]).

Anticancer peptides differ in their secondary structures; however, it is not the formal structural classes that determine functional relevance, but rather the specific motifs that enable dual modes of action. α-Helical peptides (e.g., magainins, aureins, LL-37) rapidly insert into tumor cell membranes and induce pore formation through mechanisms such as the barrel-stave and toroidal pore models. Among α-helical anticancer peptides, LL-37 (Fig. 1) is particularly notable because it combines membrane-disruptive activity with pronounced immunomodulatory functions. Structurally, LL-37 adopts a curved amphipathic helix–bend–helix conformation spanning residues 2–31, followed by a flexible, intrinsically disordered C-terminal tail. The bend within the helical region is positioned between Gly-14 and Glu-16, contributing to its dynamic membrane interactions and multifunctional biological profile (Fig. 1) [95].

**Figure 2:**
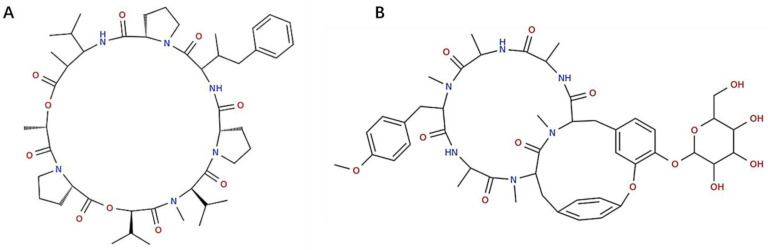
Structural representation of dolastatin 16 ((**A**), an example of cyclic dolastatins) and RA-XII (**B**).

Remarkably, LL-37 can interact with the plasma membrane of tumor cells and increase its permeability; however, its predominant anticancer activity arises from its ability to modulate the immune system. LL-37 has been shown to activate pattern-recognition receptors, particularly the Toll-like receptors TLR2 and TLR4, leading to the production of pro-inflammatory cytokines such as IL-8 and Tumor Necrosis Factor-alpha (TNF-α) and enhancing interferon-gamma (IFN-γ) mediated anticancer responses through immune-cell activation. These properties position LL-37 as a potent immunomodulatory molecule and support its therapeutic potential in combinatorial anticancer strategies rather than solely as a membrane-lytic peptide. β-Sheet peptides (e.g., defensins, lactoferricin), stabilized by disulfide bridges, exhibit high structural stability, low toxicity toward normal tissues, and a moderate yet notable immunomodulatory profile. Cyclic peptides (such as dolastatins and RA-XII, Fig. 2) represent the most structurally stable class of anticancer peptides, display nanomolar-range activity, and modulate key oncogenic signaling pathways, including phosphoinositide 3-kinase (PI3K)/Protein Kinase B (AKT)/mammalian target of rapamycin (mTOR) and nuclear factor kappa-light-chain-enhancer of activated B cells (NF-κB).

Finally, random-coil peptides (such as alloferon and PR-39) activate NK cells, induce interferon synthesis, and are important as immunomodulators in combination therapy. These structural differences determine the range of their therapeutic applications and potential for modification [96,97,98,99]. The structural features outlined above directly shape how peptides engage with specific cellular targets, thereby influencing the molecular mechanisms underlying their anticancer activity.

### 2.3 Molecular Targets and Mechanisms of Primary Interaction of Natural Peptides with Tumor Cells

Natural ACPs, despite their considerable structural and functional diversity, engage tumor cells through several conserved mechanistic patterns, dictated by the intrinsic biophysical properties of the peptides, the aberrant composition and architecture of tumor cell membranes, and the molecular dysregulation characteristic of malignant transformation. Consequently, ACPs exert multifaceted effects on cancer cells by concurrently disrupting membrane integrity, perturbing organelle function, and modulating critical intracellular signaling pathways. The following sections provide a detailed overview of the principal cellular targets underlying the primary mechanisms of ACP action [100,101].

#### 2.3.1 The Plasma Membrane Serves As the Primary Target for Interaction

The plasma membrane of tumor cells represents a major site of action for most natural anticancer peptides. Their pronounced selectivity arises from the loss of lipid asymmetry characteristic of malignant transformation. As discussed earlier, a substantial fraction of phosphatidylserine, normally confined to the inner leaflet of healthy cell membranes, becomes externalized on the outer leaflet in tumor cells. This aberrant exposure generates a markedly negative surface charge, thereby promoting strong electrostatic interactions with cationic amphiphilic peptides [102,103]. 

Heparan sulfates and sialylated glycoproteins, often overexpressed in cancer, further contribute to the increased attractiveness of the tumor membrane [104,105,106]. By interacting with these structures, ACPs can reach local concentrations sufficient to disrupt the lipid bilayer. Once bound, ACPs can trigger several membrane damage pathways: pore formation, lipid domain destabilization, microvilli disruption, changes in local membrane curvature, and lipid prolapse [107,108]. These processes lead to rapid disruption of ion homeostasis, loss of membrane potential, and a necrotic or necro-apoptotic response. Thus, the membrane acts as a “vulnerable gateway”, allowing the peptide to initiate tumor cell death before more complex intracellular mechanisms are activated [101,109].

#### 2.3.2 Mitochondria As a Target of Apoptosis

When ACPs enter the cell, they affect mitochondria, which are responsible for maintaining energy levels and controlling apoptosis. Natural peptides, such as the lactoferricin fragment LfcinB, melittin, epinecidins, and some marine peptides (e.g., paraxin), can disrupt the integrity of the mitochondrial membrane and interact with cardiolipin, a specific lipid of the inner mitochondrial membrane that is crucial for the structural organization of the respiratory chain [110,111,112]. 

The disruption of cardiolipin domains leads to a decrease in mitochondrial membrane potential, as well as the cessation of the adenosine triphosphate (ATP) synthesis, together with an increased production of ROS, and the opening of the mitochondrial permeability transition pore (mPTP) [113]. Rapid apoptosis is initiated by mitochondrial outer membrane permeabilization, which releases cytochrome c together with Smac/DIABLO, a pro-apoptotic mitochondrial protein that neutralizes inhibitor-of-apoptosis proteins and thereby amplifies caspase activation, resulting in a rapid execution of the apoptotic cascade. 

Anticancer peptides remain effective even against drug-resistant tumor cells because their activity does not depend on specific receptors or signaling components that may be lost or mutated during cancer progression. Consequently, the mitochondrial apoptotic pathway constitutes a major mechanism of ACP action, enabling selective elimination of tumor cells while exerting minimal effects on normal tissues [114].

#### 2.3.3 The Endoplasmic Reticulum As a Source of Stress and Tumor Sensitization

In recent years, it has become evident that several natural peptides can induce endoplasmic reticulum (ER) stress, a mechanism particularly associated with certain marine-derived peptides and amphibian anticancer peptides. By disrupting protein folding and impairing ER chaperone function, these peptides involve the unfolded protein response (UPR), marked by increased expression of Glucose-Regulated Protein 78/Binding Immunoglobulin Protein (GRP78/BiP), the central ER stress sensor that binds misfolded proteins; the activation of the PERK–eIF2α–ATF4 signaling axis, which reduces global protein synthesis while promoting transcription of stress-adaptive genes; and the induction of CHOP, a transcription factor that shifts the UPR toward apoptosis when stress becomes excessive. This sustained ER stress is especially significant in tumors, as prolonged UPR activation sensitizes cancer cells to additional therapeutic insults, including chemotherapy, radiotherapy, and agents that elevate reactive oxygen species. Within the tumor microenvironment, characterized by hypoxia and nutrient deprivation, these peptides accelerate cell death even further, exploiting the already compromised adaptive capacity of malignant cells [115,116]. Once these early stress responses are triggered, anticancer peptides further interfere with major survival signaling pathways, broadening the scope of their cytotoxic and regulatory effects.

#### 2.3.4 Impact on Cell Survival Signaling Pathways

In addition to directly inducing tumor cell death, ACPs can disrupt key signaling pathways that regulate malignant cell growth, survival, and adaptation. Natural cyclic peptides of marine origin, dolastatin-like compounds, and peptides derived from sponges and ascidians have been shown to inhibit the PI3K/AKT pathway, suppress NF-κB activity, and downregulate pro-angiogenic factors. Inhibition of the PI3K/AKT/mTOR axis halts cell-cycle progression and promotes both autophagy and apoptosis; suppression of NF-κB decreases the expression of inflammatory mediators and anti-apoptotic proteins; the blockade of VEGF/VEGFR-2 signaling interferes with angiogenesis, a process essential for tumor vascularization and growth; and the reduced STAT3 activation limits tumor cell migration, invasion, and immune evasion. Collectively, these signaling alterations broaden the functional repertoire of ACPs, positioning them as multifaceted anticancer agents that integrate membrane-disruptive, metabolic, and signaling-modulatory mechanisms [117].

### 2.4 Molecular and Evolutionary Pathways Underlying the Dual Activity of Natural Peptides

Many natural peptides share structural and functional features with components of the innate immune system. Their characteristic cationic charge, amphiphilic architecture, and high affinity for biological membranes enable them to exert dual biological activity: direct cytotoxic effects against tumor cells and concurrent modulation of immune responses. This dual activity stems from their evolutionary origin as host-defense molecules and from their capacity to engage conserved molecular targets, allowing them to integrate antimicrobial, immunoregulatory, and anticancer functions within a single structural framework [99,118].

Through evolution, these peptides have become universal effector elements of innate immunity. One of their key features, attracting considerable attention in oncology research, is their ability to combine a direct damaging effect on transformed cells with a pronounced immunomodulatory effect. Current research demonstrates that this dual activity has a clear molecular and evolutionary basis and reflects the fundamental principles of innate immunity [119]. Such natural peptides were developed as components of the body’s primary defense system, ensuring the rapid elimination of hazardous substances and the simultaneous activation of immune cells. Remarkably, these mechanisms also prove highly effective in the context of anticancer action.

The molecular basis for the immunomodulatory properties of ACPs is linked to their ability to interact with innate immune receptors. As mentioned in a previous section, natural peptides can activate or modulate TLR-dependent signaling pathways resulting in the release of inflammatory cytokines, activation of the transcription factors NF-κB and Interferon Regulatory Factor 3 (IRF3), and increased release of IFN-γ, interleukin (IL-18), and TNF-α [119,120]. This evolutionary background directly translates into their immunological effects, which constitute the second fundamental component of their antitumor activity.

Enhanced inflammatory and immune responses promote the activation of key components of antitumor immunity such as macrophages, dendritic cells, and natural killer cells. Among these, the influence of ACPs on NK cells is particularly significant, with peptides such as alloferon and lactoferricin enhancing NK-cell cytolytic activity by improving the recognition of stress-induced ligands on tumor cell surfaces and by increasing the production of granzymes and perforin. Restoring innate immune activity within the immunosuppressive tumor microenvironment (IME) is essential for re-establishing immune control and eliminating residual malignant cells.

In addition to modulating innate immunity, ACP-induced cytokine responses also reshape adaptive immunity. A key mechanism involves the reduction of regulatory T cells (Tregs), which normally suppresses antitumor immune responses and inhibits cytotoxic CD8^+^ T-cell function. Concurrently, the decreased secretion of immunosuppressive cytokines such as TGF-β and IL-10 renders the microenvironment more immunologically active, thereby facilitating antigen presentation and the activation of effector T cells.

From an evolutionary perspective, the dual cytolytic and immunomodulatory functions of ACPs reflect their ancestral role as innate defense molecules designed to rapidly eliminate infected or transformed cells, signal the presence of danger, and recruit immune effector populations. Thus, their direct tumor-lytic activity and their capacity to modulate immune responses represent integrated components of a unified biological strategy rather than independent mechanisms [120,121,122,123,124].

Importantly, the death of tumor cells induced by anticancer peptides can exhibit features of immunogenic cell death, a form of apoptosis characterized by the release of damage-associated molecular patterns (DAMPs) such as calreticulin, high mobility group box 1 (HMGB1), and ATP. These molecules function as endogenous danger signals: calreticulin exposure on the cell surface promotes dendritic-cell uptake of dying tumor cells, while the extracellular ATP acts as a chemoattractant and activator of antigen-presenting cells, and HMGB1 enhances antigen processing and presentation. Together, these DAMPs drive dendritic cell maturation, facilitate efficient cross-presentation of tumor antigens, and ultimately initiate a robust adaptive anticancer immune response [125,126]. This immune activation contributes to the development of long-lasting immunological memory, reducing the likelihood of tumor recurrence.

The dual anticancer activity of natural peptides arises from a complex interplay of complementary molecular mechanisms. These include direct cytotoxic effects on malignant cells, the stimulation of both innate and adaptive immune responses, the reprogramming of the tumor microenvironment, and the induction of immunogenic cell death. Together, these processes position natural peptides as multifunctional agents capable of simultaneously eliminating tumor cells and restoring effective antitumor immunity [119,126]. These characteristics distinguish anticancer peptides as a unique class of bioactive molecules capable of simultaneously suppressing tumor growth and promoting durable immune-mediated tumor control. This dual functionality is particularly relevant in the context of combination and personalized anticancer therapies, where agents that integrate direct cytotoxicity with immune modulation offer substantial therapeutic advantages.

Table 1 summarizes the most extensively studied natural peptides with demonstrated anticancer and immunomodulatory activity, detailing their biological sources, principal mechanisms of action, and current stages of preclinical or clinical development [119,120,126].

**Table 1:** Representative natural peptides exhibiting dual anticancer and immunomodulatory activity, along with key properties discussed in this work.

Peptide/Source	Molecular Weight	Anticancer Mechanisms	Immunomodulatory Effects	Tumor Types	Development Stage	Ref.
LL-37 (human cathelicidin)	~4.5 kDa (37 amino acids)	Membrane disruption; apoptosis induction; ER stress	Toll-like receptor 2 (TLR2)/4 activation; ↑ IFN-γ, interleukin-8 (IL-8); stimulation of dendritic cells and Cluster of Differentiation 8 (CD8^+^) T cells; induction of Immunogenic Cell Death (ICD)	Colorectal cancer, breast cancer, melanoma	Preclinical	[97,122,127]
β-defensins (hBD-1/2/3) (human)	3–6 kDa (30–40 amino acids)	Moderate membrane damage; inhibition of proliferation	Chemoattraction of T cells and dendritic cells; enhanced antigen presentation; tumor microenvironment remodeling	Prostate cancer, squamous cell carcinoma, colorectal cancer	Preclinical	[100,102]
Lactoferricin B (LfcinB) (lactoferrin-derived fragment)	~3.1 kDa (25 amino acids)	Membrane disruption; mitochondrial apoptosis; increased ROS generation	Enhanced Natural Killer (NK) cell cytotoxicity; reduction of Treg cells; promotion of Th1 responses	Lung cancer, breast cancer, leukemias	Preclinical	[123,127]
Melittin (bee venom)	~2.8 kDa (26 amino acids)	Rapid membrane lysis; mitochondrial depolarization	Induction of ICD; dendritic cell activation; ↑ IFN-γ	Glioma, liver cancer, breast cancer	Preclinical (nanoformulations)	[110,111]
Alloferon (insects)	~1.1–1.2 kDa (11–12 amino acids)	Limited direct cytotoxic activity	Strong activation of NK cells; ↑ IFN-γ; enhancement of immune surveillance	Melanoma, epithelial tumors	Preclinical/clinical immunomodulator	[99,127]
Magainins (amphibians)	~2.4–2.5 kDa (22–23 amino acids)	Membrane pore formation	Moderate activation of innate immunity	Skin cancer, hematological malignancies	Preclinical	[96,127]
Defensins (plant and animal origin)	~5–7 kDa (45–54 amino acids)	Membrane destabilization; growth inhibition	Stimulation of innate immune responses	Various solid tumors	Preclinical	[63,100]

Molecular weights are theoretical values calculated from amino acid sequences. Development stages are classified as follows: ‘Preclinical’ refers to peptides undergoing *in vitro* or *in vivo* validation; ‘Clinical’ refers to candidates in human Phase I–III trials. For detailed definitions and examples, see Section 6.

Most natural peptides listed here serve as biological templates and are currently in the preclinical stage, while clinical approval typically requires further structural optimization (see Section 6 for details).

### 2.5 Structural and Functional Features of Natural Peptides for the Development of New Therapeutic Approaches

In recent years, advances in peptide oncology have shifted the field from a predominantly structural classification of molecules, such as helical, β-sheet, or cyclic peptides, to a functional framework centered on their biological activities and the structural determinants that govern these effects. This functional perspective enables more accurate prediction of therapeutic potential, facilitates the rational design of targeted molecular modifications, and supports the development of optimized synthetic analogues with enhanced stability, selectivity, and efficacy [128].

The primary category comprises membrane-disrupting peptides, whose activity is governed by their amphiphilic structure, cationic charge, and capacity to form pores within the lipid bilayer. These physicochemical properties enable a rapid cytolytic effect that does not rely on specific receptors or signaling pathways on tumor cells. As a result, such peptides retain efficacy across diverse tumor types, including those exhibiting multidrug resistance, positioning them as indispensable components of modern combination therapy strategies [129,130].

The second major category consists of immunomodulatory peptides, which enhance anticancer immunity at multiple levels, ranging from the activation of NK cells and macrophages to the remodeling of the tumor microenvironment. Their structural features enable direct interaction with innate immune receptors, leading to the stimulation of cytokine production, as well as the recruitment and activation of immune effector cells, and the induction of immunogenic cell death, together creating conditions that support a robust and sustained antitumor immune response [131].

Of particular interest are peptides that affect intracellular signaling pathways, blocking key mechanisms of tumor survival and proliferation. As anticipated previously, these molecules can inhibit PI3K/AKT, (mitogen-activated protein kinase) MAPK, NF-κB, and other important pathways that determine tumor cell resistance to apoptosis [132]. Structural stability, cyclization, and the presence of hydrophobic domains or specific binding motifs enable these peptides to penetrate cells and engage intracellular target proteins, resulting in highly selective and efficient biological activity.

The fourth category consists of peptide ligands, which serve as targeted drug-delivery elements within nanomaterials by binding with high specificity to tumor-associated receptors or surface antigens, effectively functioning as molecular “targeting markers”. Incorporating such peptides into liposomes, polymeric nanoparticles, and gold- or silicon-based nanostructures markedly enhances the precision of chemotherapeutic delivery, reduces systemic toxicity, and improves the overall pharmacological profile of anticancer agents [133]. The functional classification of natural peptides reflects their true principles of action and serves as the basis for the creation of synthetic peptides, peptidomimetics, and hybrid structures capable of simultaneously combining multiple therapeutic effects. Targeted modification of the amino acid sequence, cyclization, and the introduction of D-amino acids, lipidation, or PEG modification enable control of the bioavailability, stability, and selectivity of the molecules [134].

Thus, the structural and functional analysis of natural peptides is becoming a key tool in modern peptide design. This forms the basis for the development of a new generation of anticancer drugs that combine direct cytotoxic action, immunomodulatory action, and targeted application, properties necessary for the transition of anticancer therapy to personalized and highly selective treatment strategies.

**Figure 3:**
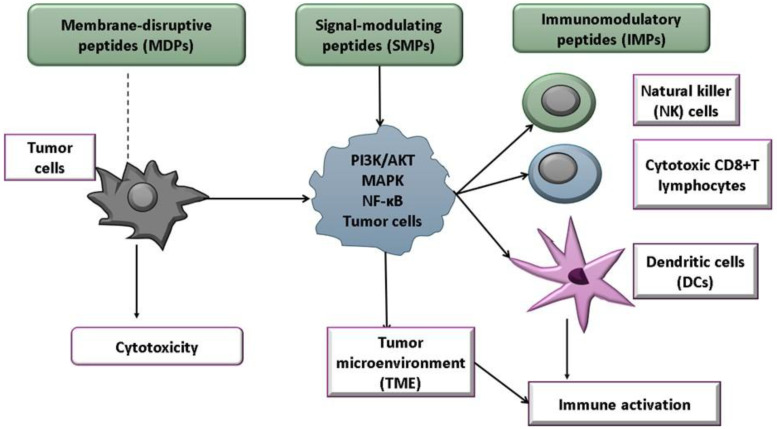
Conceptual overview of the dual anticancer and immunomodulatory activity of natural peptides. MDPs induce direct cytotoxicity through tumor membrane destabilization. SMPs regulate major oncogenic pathways (PI3K/AKT, MAPK, NF-κB), thereby affecting tumor progression and TME. IMPs enhance antitumor immunity via activation of NK cells, CD8^+^ T lymphocytes, and dendritic cells. Abbreviations: MDPs, membrane-disruptive peptides; SMPs, signal-modulating peptides; IMPs, immunomodulatory peptides; PI3K, phosphatidylinositol 3-kinase; AKT, protein kinase B; MAPK, mitogen-activated protein kinase; NF-κB, nuclear factor kappa B; NK, natural killer; DCs, dendritic cells; TME, tumor microenvironment.

For clarity, the main structural and functional categories of natural peptides and the mechanisms of their anticancer and immunomodulatory action are presented in Fig. 3, which shows how membrane-disrupting, signal-modulating, and immunomodulatory peptides simultaneously affect not only tumor cells, but also elements of the innate and adaptive immune systems, as well as certain components of the tumor microenvironment.

Direct experimental evidence suggests that specific structural motifs are precisely linked to distinct signaling cascades. For instance, the amphipathic α-helical domain of peptides like LL-37 is essential for its interaction with formyl peptide receptor-like 1 (FPRL1) and Toll-like receptors (TLRs) [97]. This interaction triggers the MAPK/ERK signaling pathway, which is responsible for promoting the chemotaxis of immune cells [97,119]. In contrast, the rigid, disulfide-stabilized β-sheet structure of defensins facilitates their binding to chemokine receptors such as CCR6, specifically leading to the recruitment of dendritic cells and memory T cells [100,102]. These mechanistic links demonstrate that the immunomodulatory outcomes of peptides are not merely consequences of membrane disruption but are governed by defined structure-activity relationships that modulate key intracellular pathways like NF-κB and PI3K/AKT [119,130].

## 3 Mechanisms of Anticancer Action of Natural Peptides

As discussed before, natural peptides exert a direct anticancer effect by altering the structure of the plasma membrane, modulating organelle function, and disrupting critical signaling pathways that control tumor cell survival [135]. These mechanisms are complex and largely depend on the tumor type, but also on factors including the presence of negatively charged surfaces, hypoxic microenvironments, mitochondrial dysfunction, membrane instability, and the compensatory overactivation of PI3K/AKT and NF-κB signaling pathways [136].

Remarkably, peptides act through mechanisms distinct from those of conventional chemotherapeutic agents, as their activity does not depend on nuclear entry or the engagement of specific cell-surface receptors. Specifically, unlike conventional chemotherapy, which primarily targets rapidly dividing cells by inducing systemic DNA damage—often leading to severe side effects such as myelosuppression and hair loss [5]—natural peptides exhibit a more selective mechanism of action. By targeting the unique biophysical properties of tumor cell membranes (e.g., negative charge and increased fluidity), peptides achieve a higher therapeutic index [7,9,10,12]. This selectivity not only reduces off-target toxicity toward healthy tissues but also minimizes the risk of secondary malignancies often associated with traditional radiotherapy and alkylating agents [5]. This receptor-independent mode of action greatly reduces the likelihood of resistance development and allows peptides to remain effective even against highly heterogeneous or drug-resistant tumor populations [136,137].

As discussed in Section 2.1, the electrostatic attraction between cationic peptides and the negatively charged surface of tumor cell membranes is the critical prerequisite for peptide accumulation. Following this initial binding to phosphatidylserine-rich and glycosylated domains, amphiphilic ACPs undergo conformational changes that allow them to insert into the lipid bilayer [138]. These processes, previously described in the section on physicochemical properties, represent the initial gateway through which anticancer peptides exert their cytotoxic effects.

Depending on the peptide structure and membrane composition, specific pore-forming mechanisms are activated, such as the “barrel-stave”, “carpet-like” or “toroidal pore” models [139,140]. All of these processes ultimately disrupt ionic homeostasis, trigger Ca^2^^+^ influx, induce rapid membrane depolarization, and culminate in cell death. This mechanism underlies the action of magainins, cecropins, lactoferricin, and some plant-derived cyclic peptides [141].

Although membrane destabilization represents one of the fastest routes to tumor cell destruction, cancer cells are largely unable to develop effective resistance to this mechanism because any attempt to alter their surface charge sufficiently to prevent peptide binding would compromise membrane integrity and overall cellular functionality [140]. Furthermore, the efficacy of these mechanisms is highly dependent on the peptide’s structural conformation. For example, while linear helical peptides are optimized for rapid membrane depolarization and pore formation [139], more structurally constrained cyclic peptides—due to their enhanced stability—are more effective at sustained interference with intracellular signaling cascades, such as the inhibition of the PI3K/AKT/mTOR axis and the suppression of NF-κB activity [135,136].

Following the initial interaction with the membrane, many peptides translocate into the cell, targeting mitochondria, which regulate energy balance and programmed cell death. Peptides such as Ra-V, dolastatin-10, several bacterial lipopeptides, and melittin induce a loss of mitochondrial membrane potential, and also provoke an increased ROS production, and cytochrome c release [142,143].

Importantly, while receptor-independent membrane disruption reduces the likelihood of classical MDR mechanisms reflecting the evolutionary plasticity of malignant systems. The apoptosis is initiated via an imbalance between Bax and Bcl-2 and the subsequent activation of caspase-9 and caspase-3. Reactive oxygen species generation is a frequent consequence of mitochondrial targeting, but it is not a universal requirement for peptide-mediated anticancer activity, as several peptides induce tumor cell death through ROS-independent mechanisms. For instance, certain membranolytic peptides like cecropins can induce rapid necrotic cell death through direct pore formation and cytosolic leakage, bypassing the requirement for intracellular ROS accumulation.

Peptides targeting mitochondria may be particularly effective against tumors with high mitochondrial instability, such as melanoma, leukemia, and glioma. Moreover, peptides can induce ER stress leading to caspase-4 activation and an enhanced pro-apoptotic response [144]. The induction of ER stress by anticancer peptides depends on peptide concentration, tumor type, and the molecular characteristics of the target cells. This dual mechanism based on the mitochondrial and ER stress amplifies the cytotoxic effect of such peptides and is accompanied by a lower risk of resistance when compared to conventional antitumor drugs.

Angiogenesis plays a critical role in tumor progression by supplying growing tissues with essential nutrients and oxygen. Since the formation of new blood vessels supports tumor expansion, sustains cellular survival, and facilitates metastatic dissemination, the inhibition of angiogenesis represents a key therapeutic strategy for restricting tumor growth and limiting disease progression. In this context, natural peptides exhibit anticancer effects by disrupting neovascularization processes, and in particular, they can inhibit VEGF/VEGFR-2 signaling pathways, reducing HIF-1α expression, and impairing endothelial cell migration [145]. These mechanisms limit microvessel growth and vascular network formation. Consequently, peptides such as KV11, PF1171A/C, and temporin-1CEa have demonstrated the capacity to inhibit tumor vascularization, thereby reducing viability and suppressing metastasis.

Another relevant biological effect of natural peptides is their ability to inhibit proteolytic enzymes, such as MMP-2 and MMP-9, which degrade the extracellular matrix and promote tumor invasion [146]. They can also disrupt cytoskeletal reorganization, reduce focal adhesions, and impede the epithelial-mesenchymal transition, thereby diminishing the tumor’s metastatic potential.

Altogether, the available evidence highlights both the considerable therapeutic potential of natural anticancer peptides and the substantial challenges that limit their direct translation into clinical practice. While the majority of naturally occurring, peptides remain confined to the preclinical stage, their structural diversity, target specificity, and broad spectrum of biological activities render them invaluable starting points for anticancer drug discovery. Importantly, progress in peptide-based oncology has largely relied on the rational optimization of natural peptide scaffolds rather than the direct use of native sequences. These observations emphasize the role of natural anticancer peptides as foundational templates for the development of clinically viable agents and provide a conceptual framework for the subsequent discussion of translational barriers and clinical advancement strategies addressed in the following sections of this review [147,148,149].

## 4 Immunomodulatory Mechanisms of Natural Peptides in Anticancer Immunity

The fundamental basis for the anticancer efficacy of natural peptides is their direct tumor cell destruction accompanied by the simultaneous immune system activation. Many natural peptides combine cytotoxic properties with alarmin functions, acting as activators of innate immunity. These peptides bind to TLR2 and TLR4 on macrophages, dendritic cells, and natural killer cells, stimulating NF-κB, MAPK, and IRF-dependent signaling pathways leading to an increased synthesis and release of pro-inflammatory cytokines, including IFN-γ, IL-12, and TNF-α. In particular, alloferon, LfcinB, and melittin are characterized by their ability to enhance NK cell cytotoxicity, stimulate macrophage phagocytic activity, and accelerate dendritic cell maturation [150]. These processes are crucial in the early stages of tumorigenesis, ensuring rapid recognition and control of transformed cells by the innate immune system.

The dual functionality of natural peptides is driven by specific physicochemical factors, primarily their net cationic charge and amphipathic secondary structure. These structural features represent the molecular basis for their bifunctional nature. The positive charge is necessary not only for the initial electrostatic attraction to anionic tumor membranes but also plays a critical role in immune recognition. In particular, experimental data show that the spatial distribution of cationic residues allows peptides to act as molecular mimics of endogenous alarmins. This allows them to bind to the negatively charged pockets of pattern recognition receptors such as TLR2 and TLR4. Following this structural interaction, the peptides trigger the recruitment of adaptor proteins (such as MyD88), which subsequently activate the MAPK and NF-κB signaling pathways. Similarly, the amphipathicity of these peptides—the segregation of hydrophobic and hydrophilic surfaces—is of critical importance; while the hydrophobic side mediates membrane insertion and pore formation, the hydrophilic residues stabilize interactions with extracellular immune receptors. Thus, the primary and secondary structures of the peptide directly determine subsequent immunological outcomes, effectively linking physical membranolysis with molecular signaling.

Natural peptides also significantly influence the adaptive immune response, as they can enhance antigen presentation by upregulating MHC-I and MHC-II molecules on dendritic cells, facilitating more effective CD8^+^ cytotoxic T lymphocyte activation. Simultaneously, the frequency and functional activity of Tregs are reduced, weakening the immunosuppressive nature of the tumor microenvironment. Some peptides also enhance T-cell chemotaxis, facilitating their infiltration into tumor tissue [151]. These combined effects not only promote stable immunological memory, but also reduce relapse risk, eventually enhancing the efficacy of immunotherapies, such as checkpoint inhibitors.

Another vital immunomodulatory property is the above-mentioned tumor microenvironment remodeling. The tumor microenvironment is often characterized by hypoxia, immunosuppression, and the accumulation of myeloid-derived suppressor cells, whose activity can be suppressed by peptides that can reduce also anti-inflammatory cytokines (IL-10, TGF-β), resulting in a shift of the tumor from an immunologically “cold” state to a “hot” immunoreactive environment [152]. Furthermore, peptide-mediated tumor cell death is frequently immunogenic, leading to the induction of ICD. During this process, the release of danger-associated molecular patterns promotes dendritic cell maturation and initiates a robust adaptive immune response [153]. Thus, natural peptides not only exert direct anticancer actions but also act as universal immune modulators, occupying a central place in next-generation therapeutic strategies in the fight against cancer. It is important to note that the direct anticancer mechanisms described above, and the immunomodulatory effects discussed in this section should not be viewed as independent processes. Peptide-induced membrane disruption, mitochondrial dysfunction, or endoplasmic reticulum stress frequently result in immunogenic cell death, thereby establishing a functional connection between direct tumor cell destruction and immune activation. During this process, the release of damage-associated molecular patterns like ATP, calreticulin, and HMGB1 helps dendritic cells mature and facilitates antigen presentation. This creates a bridge between cytotoxicity and adaptive antitumor immunity. Thus, natural peptides often exert their anticancer effects through a synergistic interaction between direct cell damage and immune activation, rather than through isolated mechanisms.

## 5 Limitations of Natural Peptides and Modern Optimization Approaches

While natural peptides hold promise in cancer therapy, their clinical application is often constrained by biological and technological factors [134]. A primary limitation is the low stability in biological environments due to the rapid proteolysis by action of serine and metalloproteases. This results in a short plasma half-life, necessitating frequent administration or protected delivery systems [154]. Moreover, their bioavailability following oral administration is typically limited by their low epithelial permeability and gastrointestinal enzymatic degradation [155]. As for their toxicity, membrane-disrupting peptides can damage normal cells at high doses, requiring careful dosage optimization and enhanced selectivity [156]. Equally importantly, the industrial-scale synthesis of complex or cyclic peptides remains challenging and costly. Additionally, inter-individual variability in immune responses can lead to heterogeneous treatment outcomes [157].

To overcome these limitations, various chemical and bioengineering strategies for structural optimization have been developed (Table 2). Amino acid substitutions allow for the regulation of charge, hydrophobicity, and secondary structure, increasing selectivity for tumor membranes [158]. The incorporation of D-amino acids and unnatural residues significantly enhances proteolytic stability and prolongs circulation [159]. Post-synthetic modifications, such as lipidation, PEGylation, and glycosylation, improve delivery, reduce clearance, and mitigate immunogenicity [160]. Cyclization and conformational stabilization strategies further improve serum stability and biological activity [161]. Chimeric and hybrid peptides also enable the combination of direct anticancer activity and immunomodulation within a single molecule (Table 2).

Another critical approach is the use of nanotechnological platforms in order to protect peptides from degradation, ensure prolonged circulation, and enable targeted delivery [162]. Since peptides can function as both active therapeutic agents and targeting ligands, such systems contribute to increase their selectivity, reduce their systemic toxicity, and may facilitate peptide blood-brain barrier penetration [163]. Finally, personalized and computational approaches are increasingly employed to optimize the properties of peptides. Bioinformatics, machine learning, and molecular modeling enable the design of synthetic “smart” peptides with predictable stability, selectivity, and endowed with properties such as anticancer action and immunological cell death induction [164]. These combinatorial approaches form the basis of a new generation of multifunctional peptide therapeutics strategies.

**Table 2:** Chemical modification strategies for optimizing the properties of natural peptides.

Type of Modification	Primary Molecular Effect	Pharmacological and Biological Consequences	Examples	Ref.
Amino acid substitutions (L → L)	Modulation of charge and hydrophobicity; stabilization of secondary structure	↑ Selectivity toward tumor membranes; ↑ cytotoxic activity; ↓ effects on normal cells	LL-37, Magainin, Temporin analogs	[134,158,160]
Incorporation of D-amino acids	Increased resistance to proteolysis	↑ Plasma half-life; ↑ serum stability; preservation of membrane activity	D-LL-37, D-Magainin	[134,159,160]
Non-natural amino acids	Conformational restriction; increased target affinity	↑ Structural stability; ↓ enzymatic degradation; improved predictability of activity	Lactoferricin analogs, cyclic peptides	[16,160]
Lipidation (palmitoylation, myristoylation)	Enhanced membrane affinity; depot formation	↑ Tumor tissue penetration; ↑ local concentration; prolonged activity	Melittin-based lipopeptides, cell-penetrating peptide (CPP) hybrids	[134,160]
PEGylation	Increased molecular weight; charge shielding	↑ Circulation time; ↓ renal clearance; ↓ immunogenicity	PEG-ACP, PEG-CPP systems	[134,160]
Glycosylation	Increased hydrophilicity; receptor-mediated interactions	↑ Bioavailability; ↑ tumor tissue selectivity; ↓ aggregation	Glycopeptide ACP analogs	[27,160]
Phosphorylation/Cholesterol conjugation	Enhanced interaction with membranes and lipid rafts	↑ Intracellular delivery; ↑ biological activity	Cholesterol-conjugated peptides	[26,160]
Cyclization (head-to-tail, disulfide bridges)	Conformational stabilization	↑ Serum stability; ↑ anticancer activity; ↓ degradation	Dolastatin-like, knottin-like peptides	[16,134,161]
Chimeric peptides (ACP + AMP)	Combination of cytotoxic and immunomodulatory properties	Dual activity: ↑ direct tumor killing + ↑ immune activation	ACP–AMP hybrids	[160]
CPP hybrids (Trans-activator of transcription (TAT), penetratin)	Active cellular transport	↑ Intracellular delivery; organelle targeting; drug delivery	CPP–ACP conjugates, nanoconjugates	[10,17,160]

In summary, the modification strategies presented in Table 2 demonstrate that chemical optimization of peptides is an effective way to overcome their limitations, improving their stability, bioavailability, selectivity, and immunomodulatory properties. This allows for the development of biologically active peptide structures closer to clinical application. These multifaceted approaches establish a strong foundation for further integration of optimized peptides into nanotechnological strategies, combination regimens, and personalized anticancer therapeutic strategies.

## 6 Clinical Statuses of Natural Peptides and Translational Challenges

Despite the significant therapeutic potential of the natural peptides discussed in this review, their successful clinical translation from the laboratory remains a formidable challenge. Most of the natural peptides are currently in the preclinical stage of development. This is largely attributed to their native origin: high susceptibility to proteolysis and low systemic bioavailability create a translational gap often referred to as the “valley of death” [163,165].

However, natural peptides serve as indispensable biological templates for the development of approved drugs. Critical analysis reveals that clinical efficacy frequently stems from the structural refinement and rational design of these naturally occurring molecules. In this context, it is important to distinguish between membrane-active ACPs, which act primarily via receptor-independent biophysical lysis, and clinical peptide-based drugs like proteasome inhibitors. Although the latter function through the specific inhibition of intracellular enzymatic complexes rather than membrane disruption, they serve as primary models for the successful structural optimization of peptide scaffolds. For instance, bortezomib and its subsequent second-generation analogs, carfilzomib and ixazomib, represent the most successful clinical milestones in peptide-based proteasome inhibition. Bortezomib, a boronic acid dipeptide, demonstrated high objective response rates in phase III trials, reaching up to 80-90% in combination therapies for multiple myeloma patients; however, its clinical utility is frequently constrained by dose-limiting peripheral neuropathy. To improve the therapeutic window, carfilzomib was developed as an irreversible inhibitor, demonstrating superior progression-free survival compared with bortezomib in head-to-head clinical trials while significantly reducing the incidence of neuropathy. Furthermore, ixazomib successfully addressed the translational barrier of oral bioavailability. As the first oral proteasome inhibitor, it offers a practical option for long-term maintenance therapy and has shown substantial benefits in extending survival outcomes, proving that rational chemical modification can overcome the pharmacokinetic limitations inherent to native peptides [147,148,163,166].

Good examples of optimization of the properties of gonadotropin-releasing hormone (GnRH) are analogs such as goserelin and leuprorelin, which are derived from the native GnRH peptide but are structurally modified to overcome the limitations of the natural sequences. Unlike endogenous GnRH, which undergoes rapid degradation, these synthetic analogues have significantly increased metabolic stability, an increased half-life, and resistance to enzymatic degradation. These properties are essential for sustained pharmacological modulation of the hypothalamic-pituitary-gonadal axis. The clinical success of goserelin and leuprorelin in hormone-dependent malignancies, including prostate and breast cancer, demonstrates that rational structural modification can transform biologically active natural peptides into therapeutically effective agents [163,167,168].

The classification of most agents in Table 1 as preclinical highlights a critical concern: a natural peptide in its native state seldom exhibits the pharmacokinetic properties requisite for immediate clinical application. Transforming a natural prototype into a viable clinical agent requires advanced strategies, such as cyclization technologies, the substitution of L-amino acids with D-enantiomers, and the utilization of nanocarriers [134,159,163].

The peptides examined in this review are classified according to their developmental phase: Preclinical (undergoing *in vitro* and/or *in vivo* validation), Clinical (optimized derivatives in Phase I–III trials, such as LTX-315 [163]), which has shown promising results in remodeling the tumor microenvironment and inducing systemic immune responses in patients with solid tumors, and Approved (peptide-based drugs with full regulatory approval, exemplified by Bortezomib [147]). Although Table 1 specifically addresses natural lead peptides, the majority of which are currently preclinical, their structural optimization has facilitated the clinical or approved status of certain derivatives. This differentiation highlights the translational gap that exists between native sequences and therapeutics suitable for clinical application. Addressing this gap requires not only structural optimization but also a shift toward multi-targeted combination regimens. Recent studies suggest that integrating peptide therapy with immune checkpoint inhibitors can significantly overcome current resistance mechanisms [169]. Furthermore, the future of peptide clinical translation is increasingly reliant on computational advancements, with the application of artificial intelligence and machine learning in smart healthcare systems providing a powerful tool for predicting clinical outcomes and personalizing peptide-based treatments [170].

## 7 Conclusion

Many natural peptides represent a unique and rapidly developing class of anticancer drugs that combine direct tumor cell damage with immune system activation. Unlike traditional chemotherapeutic drugs, they act through multi-layered mechanisms, disrupting tumor cell membrane stability, affecting mitochondrial function, inhibiting important tumor signaling pathways, and simultaneously activating innate and adaptive immune responses. This combination of biological effects allows peptides to overcome classical drug resistance mechanisms and promote immunological activation of the tumor microenvironment. In fact, therapeutically-relevant properties including the induction of immunogenic tumor cell death, together with the enhanced antigen presentation, the activation of NK cells and cytotoxic T lymphocytes, as well as the suppression of immunosuppressive cell populations by natural peptides, all contribute to the formation of long-term anticancer immune memory. Thus, these observations support the concept of integrated peptide-mediated immune and membrane modulation as a holistic conceptual framework. According to this model, natural peptides exert a co-ordinated action selectively destabilizing tumor cell membranes while simultaneously reorganizing the immune microenvironment to enhance innate and adaptive antitumor responses. According to this model, natural peptides exert a concerted action selectively destabilizing tumor cell membranes while simultaneously reorganizing the immune microenvironment to enhance innate and adaptive antitumor responses. This integrated perspective suggests that the cytotoxic and immunomodulatory activities of natural peptides are not separate mechanisms but components of a unified evolutionary strategy, offering a conceptual framework for the development of next-generation peptide-based anticancer therapies. Furthermore, structural optimization and chemical modification of peptides, as well as nanotechnological delivery systems developed in recent years, have significantly increased their stability, bioavailability, and clinical applicability, enabling more targeted and safer therapeutic applications. At the same time, the integration of bioinformatics, computational design, and personalized medicine in peptide-based oncotherapy opens new opportunities for the creation of “smart” peptides with high selectivity and predictable biological profiles, enabling the integration of direct anticancer action and immunomodulation within a single therapeutic platform, which thereby increases treatment efficacy and reduces systemic toxicity. Overall, natural peptides represent a biological basis for the development of a new generation of anticancer drugs that combine pronounced cytotoxic activity and powerful immunomodulatory potential. Their in-depth structural and functional study, rational optimization of their structures, the integration of nanotechnology solutions, and the development of personalized approaches are paving the way for a transition to highly selective, minimally invasive, and immunologically effective cancer treatment strategies in the coming years.

Importantly, the clinical translation of peptide-based anticancer strategies is closely linked to the development of personalized medicine frameworks. In this context, patient-specific biological and immunological parameters, including tumor membrane characteristics, immune competence, and systemic inflammatory status, represent realistic determinants of therapeutic responsiveness. The multidimensional nature of these factors highlights the limitations of conventional analytical approaches. In the context of clinical practice, the most realistic parameters for guiding personalized peptide selection include: (1) the tumor’s membrane lipid profile (specifically the degree of phosphatidylserine exposure), (2) the acidity (pH levels) of the tumor microenvironment, as many anticancer peptides are pH-dependent, and (3) the local expression of specific proteases, which determines the peptide’s metabolic stability in a particular patient. Additionally, characterizing the patient’s immune landscape, such as the density of tumor-infiltrating lymphocytes (TILs), can help identify those who would benefit most from the immunomodulatory action of these peptides. Moreover, artificial intelligence and machine learning provide the critical link between patient-specific data and personalized peptide therapy by enabling the integration and interpretation of complex biological, immunological, and clinical datasets. Rather than functioning as independent concepts, AI-driven tools serve as decision-support systems that translate patient-level information into rational peptide selection and optimization strategies. This synergy between computational approaches and personalized medicine facilitates the development of adaptive, “smart” peptide-based anticancer therapies that combine direct cytotoxic activity with immunomodulatory effects, ultimately enhancing treatment efficacy while minimizing systemic toxicity.

## Data Availability

Not applicable.
